# Comparison of the effectiveness of integrative immunomodulatory treatments and conventional therapies on the survival of selected gastrointestinal cancer patients

**DOI:** 10.1038/s41598-023-47802-5

**Published:** 2023-11-21

**Authors:** Ralf Kleef, Magdolna Dank, Magdolna Herold, Emese Irma Agoston, Julia Lohinszky, Emoke Martinek, Zoltan Herold, Attila Marcell Szasz

**Affiliations:** 1 Dr. Kleef Medical Center, 1030 Vienna, Austria; 2 Division of Oncology, Department of Internal Medicine and Oncology, Semmelweis University, Budapest, 1082 Hungary; 3 Department of Internal Medicine and Hematology, Semmelweis University, Budapest, 1088 Hungary; 4 Department of Surgery, Transplantation and Gastroenterology, Semmelweis University, Budapest, 1082 Hungary

**Keywords:** Oncology, Cancer

## Abstract

In the last decade, the use of immunomodulating treatments (IMT) at integrative oncology providers (IOP) increased. IMTs are used to modulate the tumor microenvironment, which might lead to increased response-to-treatment, and the indication of immune checkpoint inhibitors might also be widened. The efficacy and safety of IMTs in advanced/metastatic gastrointestinal cancers were compared with conventional chemo(radio)therapy (CT). 21 colorectal– (CRC), 14 pancreatic– (PC), 5 cholangiocellular– (CCC), 5 gastric– (GC) and 4 esophageal cancer (EC) patients received IMT. IMT and CT were compared in CRC and PC. CT was administered at an academic oncology center. After the initiation of IMT, a median survival of ~ 20 (CRC, PC and EC) and ~ 10 months (CCC and GC) was observed. Of the IMTs, locoregional modulated electro-hyperthermia had the most positive effect on overall survival (HR: 0.3055; *P* = 0.0260), while fever-inducing interleukin-2, and low-dose ipilimumab showed a positive tendency. IMT was superior to CT in PC (HR: 0.1974; *P* = 0.0013), while modest effect was detected in CRC (HR: 0.7797; *P* = 0.4710). When the whole study population was analyzed, IMTs showed minimal effect on patient survival, still CT had the greatest effect if introduced as early as possible (HR: 0.0624; *P* < 0.0001). The integrative IMTs in the presented form have mild impact on gastrointestinal cancer patients’ survival, however, we observed its benefit in PC, which warrants further investigations.

## Introduction

Gastrointestinal cancers account for 25.8% and 35.4% of all new cancer cases and cancer-related deaths, respectively^1^. To date, surgical resection (if possible) and chemotherapy/chemoradiotherapy and/or biological/targeted therapies are the gold standards for their treatment, however, despite all the efforts in the latest drug development and state-of-the-art surgical methods, the 5-year survival rate of advanced stage gastrointestinal cancers is still under 15%^2^. In the last decades, a new trend in the treatment of cancers has emerged that takes advantage of the immune system and fights cancer by reactivating the body’s natural immunity. It is suggested, that based on the amount of T-cell-infiltration, immunologically “cold” and “hot” tumors can be distinguished^3^. “Hot” tumors are known to have a high level of intratumoral infiltrating T-cells, a high level of tumor mutational burden, and a less immunosuppressive tumor microenvironment. In contrast, “cold” tumors have a low level or no T-cell infiltration at all, low tumor mutational burden, and a highly immunosuppressive tumor microenvironment^3–7^. The immunosuppression largely occurs through immune checkpoint molecules, which are expressed by the tumor itself helping it escape the immune surveillance mechanisms^2^. The majority of gastrointestinal cancers are known to be “cold” tumors^2–4^, and only a very limited number with specific phenotypes are “hot” and respond well to immune checkpoint inhibitor (ICI) therapy^3^. Immunotherapy using ICIs in the “hot” subtypes is part of the standard of care. Moreover, numerous attempts have been made lately to make cancer of different origins immunologically “hotter”, which may result in an overall better response to treatment, and in the wider applicability of ICIs^6–9^. In vitro cellular and in vivo animal research have found that artesunate^10^, curcumin^11^, dichloroacetate^12^, high-dose vitamin C^13^, interferon-γ^14^, interleukin-2^15^, ozone therapy^16^, and various forms of oncological hyperthermia^17,18^, including whole-body hyperthermia (WBH)^17^ and modulated electro-hyperthermia (mEHT)^18^, can modulate the immune system and induce antitumoral mechanisms. Although, data on their clinical utility is very limited, it is important to understand the basis of the off-label use of these products. Therefore, the available clinical data on them is reviewed in the Supplementary Materials of this article. It has to be emphasized, that, to our knowledge, several integrative oncology providers (IOP^19^) offer such therapies in various combinations, however, no literature data is available about these applications.

Although, in conventional oncology treatment centers none of these off-label treatment modalities are available, an emerging number of IOPs^19^ offer patients these treatment modalities, despite the lack of safety and/or efficacy data from randomized or observational studies. Uniquely, we have published very promising results about an integrated immunomodulatory treatment (IMT) method previously^20^. However, to our knowledge, no further result(s) emerged in the literature since. Therefore, a retrospective pilot study was conducted in collaboration between an IOP (Dr. Kleef Medical Center, Vienna, Austria) and an academic oncology center (Semmelweis University, Budapest, Hungary) to collect data about the safety and efficacy of IMT, compared to conventional treatments. Further research questions included the direct comparison of the two approaches and attempts were made to identify their benefits and caveats.

## Results

**Figure 1:**
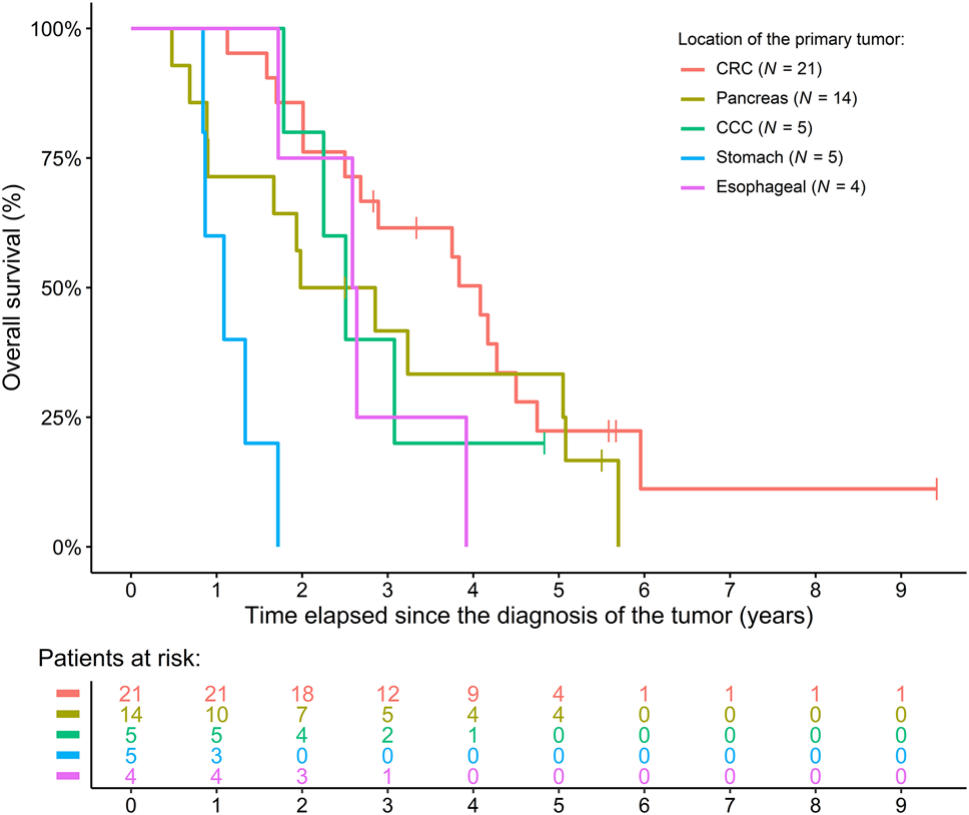
Overall survival of the five tumor cohorts treated with immunomodulatory treatments. CCC: cholangiocellular cancer; CRC: colorectal cancer.

Of the forty-nine gastrointestinal cancer patients who received IMT, 21 (42.86%) had colorectal cancer (CRC), 14 (28.57%) had pancreatic cancer (PC), 5 (10.20%) had cholangiocellular cancer (CCC), 5 (10.20%) had gastric cancer (GC), and 4 (8.16%) had esophageal cancer (EC). Those patients developing CRC had the highest probability of having the primary tumor removed, while, as expected, at least half of the patients had inoperable CCC, EC, GC, and PC. 18 (85.7%) of the 21 CRC, 9 (64.3%) of the 14 PC, 3 (60%) of the 5 CCC, 2 (40%) of the 5 GC, and 3 (75%) of the 4 EC patients received conventional chemo(radio)therapy (CT) prior IMT. The time between the diagnosis of the tumor and the initiation of IMT was the longest in CRC (23.08 ± 20.15 months), followed by CCC, PC, EC, and the shortest was in GC (4.02 ± 3.56 months). At the time of IMT initiation, most of the patients developed distant metastases. A 49-, 29-, 30-, 13-, and 31-month-long median survival for CRC, PC, CCC, GC, and EC was observed, respectively, if survival was calculated from the diagnosis of the tumor (Fig. 1). After the initiation of IMT, median survival was similar in CRC (20.01 months), EC (21.82 months), and PC (17.91 months), while shorter survivals could be observed in CCC (11.53 months) and GC (9.00 months). Detailed anamnestic and clinicopathological data of the five tumor cohorts can be read in Table 1.

**Table 1:** Anamnestic and survival data of patients receiving immunomodulating therapy (IMT). Unless otherwise indicated, continuous and count data are presented as the mean ± standard deviation and the number of observations, respectively.

Parameter	CRC (N = 21)	Pancreatic (N = 14)	CCC (N = 5)	Gastric (N = 5)	Esophageal (N = 4)
Age (year)	49.00 ± 15.68	55.83 ± 9.61	53.96 ± 14.44	54.74 ± 13.72	52.02 ± 11.93
Sex (male : female)	7 : 14	5 : 9	2:3	1:4	0:4
Body-mass index (kg/m^2^)	22.21 ± 3.62	22.60 ± 4.61	23.80 ± 1.82	22.36 ± 6.64	22.76 ± 2.76
Primary tumor					
Completely / partially resected	19/0	7/0	1/0	1/1	1/0
Inoperable or not operated	2	7	4	3	3
TNM^40^					
T: 1/2/3/4/irresectable	2/2/8/7/2	0/3/2/2/7	0/1/0/0/4	1/0/0/0/4	0/0/0/1/3
N: 0/1/2/irresectable	3/7/9/2	4/3/0/7	1/0/0/4	0/0/1/4	0/0/1/3
Metastasis: synchronous / metachronous	11/9	8/5	3/1	4/1	2/2
Location of metastases					
Liver	12	7	3	1	2
Lung	8	4	1	1	2
Distant lymph nodes	9	1	3	2	2
Peritoneal	7	3	0	3	0
Ovarium	3	1	0	1	0
Locoregional	4	3	0	0	0
Other (e.g., bone, brain)	0	2	1	3	0
Medical history prior cancer diagnosis					
Hypertension	4	3	2	0	0
Diabetes mellitus	4	0	0	0	0
Stroke / MI / other CV diseases ^1^	0/3/3	0/0/2	0/0/3	0/0/0	0/0/0
Thyroid disease	4	2	1	0	0
Autoimmune disease	4	0	1	0	0
Time to first IMT (month)	23.08 ± 20.15	12.53 ± 14.07	19.69 ± 10.69	4.02 ± 3.56	13.03 ± 10.31
Median survival time since the					
Tumor diagnosis (month)	48.99	28.98	30.09	13.01	31.34
1^st^ day of IMT (month)	20.01	17.91	11.53	9.00	21.82
Last day of IMT (month)	15.41	9.31	10.51	1.15	5.73
Survival rate since 1^st^ IMT					
1-year (%)	57.1	64.3	40.0	20.0	75.0
2-year (%)	41.9	21.4	20.0	0	25.0
3-year (%)	27.9	0	0	0	0

^1^Other CV diseases included, e.g., congestive heart failure, atrial fibrillation, peripheral artery disease, etc. CCC: cholangiocellular cancer; CRC: colorectal cancer; CV: cardiovascular disease; MI: myocardial infarction, TNM: Tumor – Node – Metastasis staging system.

Of the IMTs, locoregional mEHT (median: 16; range: 2–51) and high-dose vitamin C treatment (median: 15; range: 2–38) were used the most often, followed by low-dose ICIs (median: 3; range: 1–12) and WBH (median: 6; range: 1–25) treatments. IMT sessions are summarized in Table 2. The effect of the IMT modalities on patient survival was assessed, using an extended Cox model, where the IMTs were included as time-dependent coefficients, and different baseline hazards were assumed for the five tumor cohorts. This modeling approach was necessary due to the different combinations and initiation times of the IMTs. In addition, a few patients also received CT alongside (CRC: 5, PC: 3, CCC: 2, GC: 2, EC: 0) or after (CRC: 4, PC: 7, CCC: 4, GC: 2, EC: 0) the IMT. Therefore, it was also added as a possible effector of patient survival. Model results showed that locoregional mEHT (HR: 0.3055; 95% CI 0.1075–0.8680; *P* = 0.0260) and CT (HR: 0.2688; 95% CI 0.0873–0.8280; *P* = 0.0221) had a significantly positive effect on patient survival, while fever-inducing interleukin-2 had tendentially positive effect (HR: 0.2212; 95% CI 0.0402–1.2150; *P* = 0.0826). Furthermore, a second model was also created, where the low-dose ICIs were not investigated together, but as separate model predictors. In addition to the previously detailed effects of mEHT, CT and interleukin-2, the use of ipilimumab was also associated with tendentiously longer survival times (HR: 0.0720; 95% CI 0.0038–1.3620; *P* = 0.0794).

**Table 2:** Number of immunomodulating therapy sessions. Data are presented as mean (range).

Therapy	CRC (N = 21)	Pancreatic (N = 14)	CCC (N = 5)	Gastric (N = 5)	Esophageal (N = 4)
Locoregional mEHT	16.57 (6–42)	19.71 (2–47)	20.20 (13–35)	25.40 (13–51)	16.25 (5–44)
wIRA hyperthermia	2.10 (0–19)	1.93 (0–16)	2.60 (0–13)	5.80 (0–17)	0.25 (0–1)
Mild WBH	3.52 (0–8)	4.43 (1–8)	4.80 (3–7)	5.60 (0–15)	5.25 (2–10)
Moderate WBH	1.71 (0–15)	1.79 (0–13)	2.60 (0–9)	0.00 (0–0)	0.25 (0–1)
Long duration moderate WBH	1.00 (0–3)	2.14 (1–12)	1.20 (1–2)	2.60 (1–4)	1.00 (0–2)
Low-dose ipilimumab ^1^	3.57 (0–12)	3.36 (1–6)	4.20 (3–6)	5.00 (3–12)	6.00 (3–9)
Low-dose nivolumab ^1^	4.09 (1–12)	3.43 (1–7)	3.60 (2–6)	6.60 (3–12)	6.00 (3–9)
Interleukin-2	3.86 (1–22)	3.93 (1–14)	7.40 (1–18)	2.80 (1–5)	3.25 (1–6)
High-dose vitamin C	14.81 (2–35)	16.86 (4–38)	19.40 (13–36)	22.20 (13–34)	15.00 (5–37)
Curcumin	2.00 (0–21)	1.14 (0–12)	4.20 (0–15)	1.00 (0–5)	2.50 (0–10)
Dichloroacetate	1.71 (0–8)	3.14 (0–22)	5.40 (0–12)	1.60 (0–4)	4.00 (0–13)
Artesunate	2.05 (0–8)	0.86 (0–8)	5.20 (0–11)	1.00 (0–5)	0.00 (0–5)
Ozone therapy	0.81 (0–4)	1.07 (0–7)	2.40 (0–5)	0.00 (0–0)	0.50 (0–2)
Interferon-γ	1.57 (0–13)	2.64 (0–13)	3.80 (0–9)	1.00 (0–5)	0.00 (0–0)
Metronomic chemotherapy	0.57 (0–11)	3.93 (0–15)	1.20 (0–3)	5.40 (0–18)	2.75 (1–5)

^1^A single patient in the CRC cohort received nivolumab as monotherapy. CCC: cholangiocellular cancer; CRC: colorectal cancer; mEHT: modulated electro-hyperthermia; WBH: whole-body hyperthermia; wIRA: water-filtered infrared-A.

It was also investigated whether the addition of further clinicopathological characteristics into the survival models as explanatory parameters influences on the IMTs. The survival models described in the previous paragraphs were extended with the following: age, sex, when the metastasis occurred (none / metachronous / synchronous), and the location of the metastases (liver, distant lymph node, peritoneal and/or locoregional). In both cases, the effect of peritoneal metastases on patient survival was too large, therefore it was excluded from the final models. In the model, where the two ICIs were investigated together, mEHT (HR: 0.4122; 95% CI 0.1279–1.3282; *P* = 0.1377) and CT (HR: 0.2368; 95% CI 0.0641–0.8755; *P* = 0.0308) was tendentiously and significantly associated with longer survival times. Neither the other IMTs, nor the newly introduced clinicopathological parameters had any effect on patient survival. Similarly, where ipilimumab and nivolumab were investigated separately, mEHT (HR: 0.3388; 95% CI 0.1005–1.1424; *P* = 0.0809), ipilimumab (HR: 0.0697; 95% CI 0.0030–1.5956; *P* = 0.0954) and IL-2 (HR: 0.1840; 95% CI 0.0264–1.2814; *P* = 0.0873) had tendentiously, and CT (HR: 0.2640; 95% CI 0.0737–0.9464; *P* = 0.0409) had significantly positive effect.

The most common adverse events (AEs) related to the IMTs were fever with chills, rashes, vomitus, weakness, malaise, neutropenia, and hyperthermia-related local skin reactions: skin redness and grade I burns. Less common AEs included sleeping problems, limb edema, elevated liver enzymes, and diarrhea. In a single case, bloody stool without further occurrence was observed after the first administration of fever-inducing interleukin-2. A total of three serious AEs occurred: (i) Reversible kidney failure due to ICI-associated nephritis^21^, (ii) bleeding of a rectal tumor requiring hospitalization after the first administration of fever-inducing interleukin-2, and (iii) psoriasis exacerbation.

### Comparison of CRC patients treated with conventional and IMT therapy

The 21 IMT-treated CRC patients were compared to age, sex, TNM staging, tumor location, and metastasis occurrence time (none, synchronous, metachronous) matched cohorts, who received only CT. As the CT cohort was significantly larger, namely 835 patients, in addition to 1:1 matching, it was also feasible to perform 2:1, 5:1 and 10:1 matching. The inclusion of higher matching ratios into the analysis was done to reduce selection bias. Clinicopathological data and comparison results of the IMT and matched CRC cohorts are summarized in Tables S1 and S2. Except for the following, all cohorts were basically identical. In the 1:10 pairing, the non-IMT patients were older than their IMT pairs (IMT: 49.00 ± 15.68; non-IMT: 59.48 ± 10.94; *P* = 0.0067).

**Figure 2:**
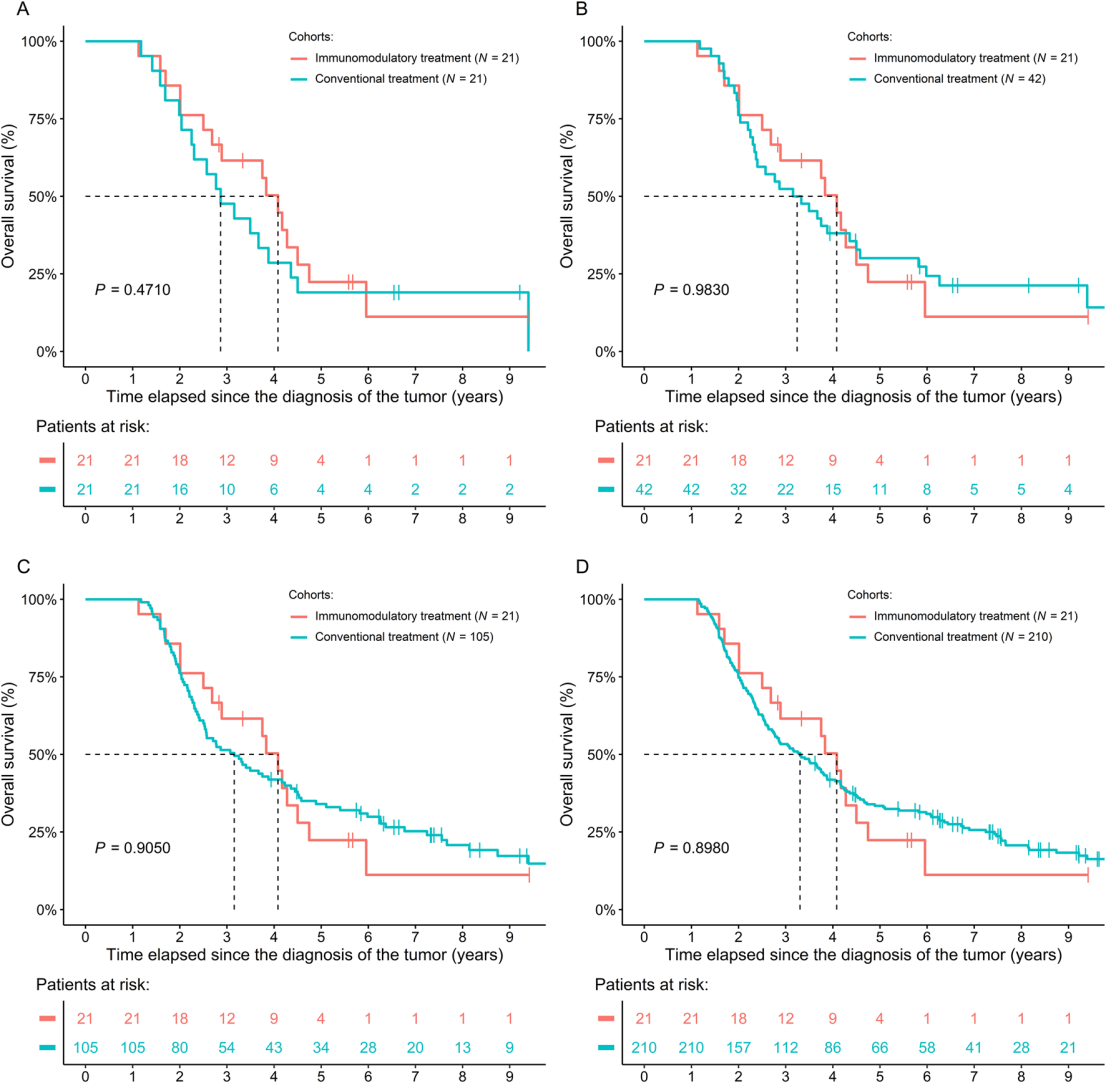
Comparison of survival data of colorectal cancer patients treated with and without immunomodulatory treatment (IMT) modalities. The IMT cohort was matched with a significantly larger (N = 835) cohort of patients having only conventional chemo(radio)therapy, therefore, it was feasible to investigate the differences in (**A**) 1:1, (**B**) 2:1, (**C**) 5:1, and (**D**) in 10:1 matched cohorts.

Survival analysis revealed no difference between the IMT and 1:1 matched cohorts (*P* = 0.4710; Fig. 2A), and similar results were found for the 2:1 (*P* = 0.9830; Fig. 2B), 5:1 (*P* = 0.9050; Fig. 2C), and 10:1 (*P* = 0.8980; Fig. 2D) cohorts. If analyzed in a time-dependent manner, neither the effect of all (*P* = 0.4650) nor of individual IMTs could be verified, while the early introduction of CT within the course of the disease was the most effective predictor of longer patient survival (HR: 0.0779; 95% CI 0.0290–0.2090; *P* < 0.0001). All model results stayed the same, even if age, sex, and metastasis occurrence were added to the models.

### Comparison of PC patients treated with conventional and IMT therapy

Three cohorts were compared: the 14 IMT-treated PC patients, and 14–14 age, sex, tumor location, and metastasis occurrence time (none, synchronous, metachronous) matched PC patients, who were treated with CT with or without concomitant mEHT. Clinicopathological results and their comparison of the three PC cohorts are summarized in Tables S3 and S4. It has to be noted, that in the IMT-treated cohort the number of patients with an irresectable PC was less common, compared to that of the control cohorts. Therefore, in all subsequent analyses, all models were corrected for this parameter.

**Figure 3:**
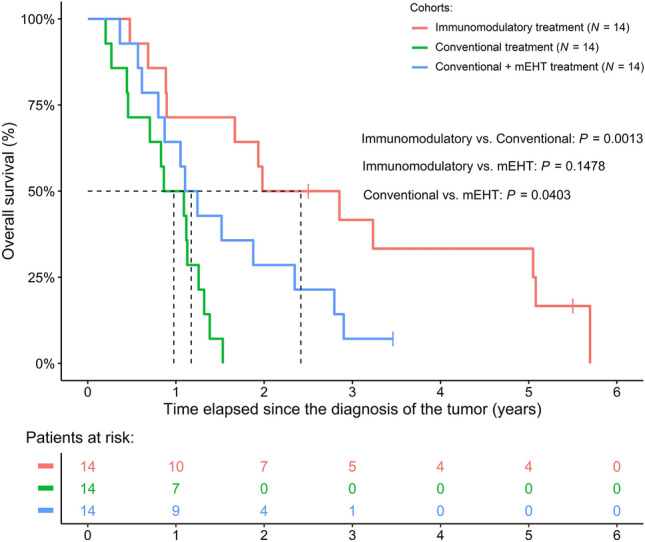
Survival difference of pancreas cancer patients between the immunomodulatory treatment and matched control cohorts with and without modulated electro-hyperthermia (mEHT) treatment. It has to be noted that naïve Kaplan–Meier curves were drawn on the figure, while the *P*-values were obtained using baseline hazard adjusted Cox regression models. Baseline hazard adjustment was performed due to the fact, that in the “Conventional” and “Conventional + mEHT” cohort the number of patients with inoperable pancreatic tumors were higher.

The IMT cohort had longer survival than the CT (reduced risk of the IMT cohort vs. CT: HR: 0.1974; 95% CI 0.0736–0.5295; *P* = 0.0013), but had the same survival as the CT + mEHT cohort (IMT vs. CT + mEHT: HR: 0.4892; 95% CI 0.1858–1.2880; *P* = 0.1478). The shortest survival was found in the CT-only cohort (survival advantage of the CT + mEHT cohort vs. CT only: HR: 0.4035; 95% CI 0.1695–0.9606; *P* = 0.0403). The naïve Kaplan–Meier curves of the three cohorts are drawn on Fig. 3. When analyzing the various IMT options and their effect on patient survival separately, mEHT had tendentious effect on the survival of PC patients (HR: 0.1082; 95% CI 0.0098–1.1954; *P* = 0.0696). Extending the models with age, sex, and metastasis occurrence time yielded consistent results. None of the additional clinicopathological parameters had any effect on survival, and the same tendencies regarding the patient cohorts [IMT (ref.) vs. CT: *P* = 0.0007; IMT (ref.) vs. CT + mEHT: *P* = 0.0794; CT (ref.) vs. CT + mEHT: *P* = 0.0372] and mEHT (*P* = 0.0546) could be justified in every extended model.

### What effect does IMT have on patient survival?

As detailed above, most IMT-treated patients received CT during the course of their disease: either prior, alongside or after IMT. Therefore, it was possible to test the effect of IMT and CT on patients’ survival as follows. We chose two approaches, first, a simplified time-dependent extended Cox survival model with adjusted baseline hazards for the different tumor cohorts was analyzed, in which we specified the intervals when the patients received either CT, IMT, simultaneously both, or no therapy at all. Results showed that IMT in general had no effect on patient survival neither if introduced close to cancer diagnosis (*P* = 0.9980), nor if used in a later stage of the disease (*P* = 0.8350). In contrast, CT had the best positive effect on patient survival if it was administered shortly after tumor diagnosis (HR: 0.0624; 95% CI 0.0193–0.2022; *P* < 0.0001), while its late introduction/use had a less obvious effect (*P* = 0.5710).

Second, the individual therapeutic options were analyzed after tumor diagnosis. We could justify the positive significant effect of mEHT (HR: 0.1984; 95% CI 0.0558–0.7051; *P* = 0.0124) and CT (HR: 0.0895; 95% CI 0.0262–0.3063; *P* = 0.0001). Moreover, metronomic chemotherapy had a marginal association with longer survival times (HR: 0.2244; 95% CI 0.4985–1.0100; *P* = 0.0516). None of the remaining IMTs showed any effect.

If the two models described in the above paragraphs were extended with further, clinicopathological parameters, the following was found. In the extended model, where we investigated the effect of combined IMT, CT, age, sex, and metastasis data, the same was observed as above. IMTs had slight effect over patient survival: neither if introduced closer to the cancer diagnosis (*P* = 0.9988), nor if introduced at a later time (*P* = 0.9552). CT had the most positive effect if introduced early (HR: 0.0395; 95% CI 0.0080–0.1947; *P* < 0.0001), and older age of the patients (HR: 1.0440; 95% CI 1.0030–1.0880; *P* = 0.0351) was also a significant predictor. Similarly, the second model also predicted the same as before: mEHT (HR: 0.2496; 95% CI 0.0569–1.0960; *P* = 0.0659), CT (HR: 0.0817; 95% CI 0.0206–0.3240; *P* = 0.0004) and metronomic chemotherapy (HR: 0.2162; 95% CI 0.0408–1.1460; *P* = 0.0718) being tendentious/significant effectors.

### Additional observations

We could identify the following additional information and trends from the documentation of the IMT-treated patients: The socioeconomic status of these patients was high. Long-lasting stable disease was observed in several cases. It should be noted, however, that we could not identify a standardized protocol for the selection of treatment options and the later introduction of additional treatments seemed also random. Moreover, compared to the strict schedule of CT, the various IMT treatments could often be postponed based on the preference of patients, and the diagnostic tests used for treatment selection often had no normal values or acceptable standards.

## Discussion

Treating advanced-stage cancer is a challenging task for the medical staff, patients, their families and the society at large. Compared to conventional oncology centers, which operate on strict guidelines and evidence-based treatment options only, IOPs offer a more patient-accepted approach^22^. In addition to dietary supplements, herbal medicines, and traditional treatments, the use of off-label drugs is also very common^19,22^. Although more and more IOPs offer some kind of immunotherapy^19,23,24^, and in previous research we could have demonstrated improved patient survival and curative effects in stage IV cancer patients^20^, to our knowledge, this is the first report in gastrointestinal tumors that analyzed and compared integrative IMTs to conventional oncological treatments.

A large set of literature data supports that these—in most cases off-label—therapeutic solutions have some effect in the treatment of gastrointestinal tumors on their own^10–18^, however, the lack of randomized clinical trials and/or evidence-based data prevents their use in CT. In contrast, IOPs around the world often offer these drugs either standalone or in various combinations. In the current study we found that of the administered IMTs, mEHT evidently had a significant effect on patients’ survival, which was comparable to that of CT, followed by the promising, tendentiously positive effect of interleukin-2 induced artificial fever therapy and low-dose checkpoint inhibitors (ipilimumab + nivolumab). However, we were not able to demonstrate sufficient certainty about the other IMT modalities that their usage significantly promotes longer survival of patients, while CT had a strong positive effect on patient survival in all of the analyses we performed. The same result was obtained in those models where the analysis was “simplified” to compare CT and IMTs in general: mild effect of IMTs could be observed, while CT is the most effective if it is introduced after the diagnosis of the tumor as early as possible. Similarly, no effect of IMT on survival was found when the CRC cohort was investigated separately, even with varying matching ratios, while the superiority of the IMT approach over CT without mEHT was found in PC, but the same survival was found when CT was supported by concomitant mEHT. No statistical difference was observed between the survival of the IMT and CT + mEHT cohorts. The number of PC patients included in the present study was relatively low, but besides the differences in the treatment, we could not prove any other effector that could cause this difference. We hypothesize a possible strong effect of the combined use of mEHT, ipilimumab and nivolumab, however, the data in the literature about ipilimumab and nivolumab in PC are controversial and scarce^25–28^. Therefore, this observation needs further investigation as soon as possible.

It was also investigated whether the extension of the survival models with further clinicopathological parameters changed the above-detailed results. The same results could have been justified, even if known strong predictors, such as age, presence / development time of metastases, etc. were included. All these consistencies ultimately strengthen that CT is the most important factor in a cancer patient’s survival, and of the IMTs mEHT, fever-inducing interleukin-2 and low-dose ICIs seem to be those, that need further investigations. In the literature, most data is available about mEHT: e.g., the studies investigating mEHT in PC have found the same positive tendencies over patient survival^29–33^ as detailed in the results of the current study. Therefore, it is strongly suggested to further investigate the positive effects of concomitant mEHT in (gastrointestinal) cancer.

As detailed in the previous paragraphs, except for mEHT, IMTs had no statistically justifiable effect on patients’ survival. Based on the data collected, we can only speculate the reason behind this observation. During the course of the disease, the various types of IMTs were introduced randomly, sometimes closer to the diagnosis, while in other cases significantly, even years later. Moreover, the various drugs were often used with low doses, and their application was often based on whether the patient could afford it or not. These are some of those biases, that we hypothesize might have significantly affected the efficacy of these therapeutic options. Therefore, systematic, multicenter research conducted in the future would be ideal to further investigate the effect of these therapeutic options. Furthermore, we believe, the strong advantage of CT, which was found in the current research, might also arise from these biases of the IOPs. During the administration of CTs the medical staff needs to follow strict rules, and all therapeutic decisions are made based on evidence-based tools, tests, etc. While, in the philosophy of IOPs, the patient's decision/preference comes first. All of these points to that the introduction of standardized protocols is essential and necessary in the case of IOPs as well.

The following side effects and adverse events were registered throughout the study. Local skin reactions (redness and grad I burns) were mostly associated with mEHT, while weakness, malaise, and sometimes fever were associated with WBH, in line with previous descriptions^34^. Similarly, the occurrence of fever with chills, rashes, vomiting, neutropenia, sleeping problems, limb edema, elevated liver enzymes, and diarrhea are known frequent side effects of the drugs used during IMT^35–38^. In addition, three previously described serious AEs occurred, namely, an ICI associated nephritis^21^, a psoriasis exacerbation manageable using standard psoriasis treatments^39^, and a bleeding of a rectal tumor requiring hospitalization after the first administration of fever-inducing interleukin-2^40^. It can be determined that there is a strong relationship between the described serious AEs and the applied treatments. As all serious AEs were known from the literature and particular attention was paid to known signs and symptoms. In the article of Belliere et al.^21^, it was suggested that renal monitoring of all patients receiving ICI treatments is necessary. In the current study, regular blood samples were taken before, during and after the treatments, including creatinine and eGFR testing. Similarly, psoriasis flares manageable with standard treatments^39^ and grade 3 or 4 hemorrhages^40^ are known side effects of ICIs and interleukin-2, respectively. Although in all cases, the serious AEs resolved without sequelae, it is important to draw attention to the fact that the treatment of serious AEs always requires hospitalization, often at a different, external institution. Most IOP centers themselves are not sufficiently prepared to handle serious AEs, as these events require specialized tools, wards, and personnel, such as an intensive-care unit. Furthermore, comorbidities, such as autoimmune diseases make oncology treatments difficult even in traditional centers, therefore, treating such patients at IOPs requires extra attention and/or the involvement of specialist(s). On the basis of the AE cases processed during our research, for the protection of patients, it is extremely important for every IOP to have an emergency cooperation partner. We recommend that during these collaborations, the partner healthcare institution should be adequately informed about the treatments and drugs used at the IOP, that way they can adequately prepare for the care of these patients and (serious) AE events.

Based on the observations of the current study, the main shortcoming of IOPs is the lack of guidelines, followed by the excessive influence of patients on therapeutic decision processes, socioeconomic considerations, and the lack of public and/or private health insurance support for the applied therapies, which has also been criticized by others before^19,41^. The problem of the lack of guideline(s) was evident at the very beginning of our research. As described in “Results”, even though we had access to the complete treatment data, we were unable to define a uniform protocol. This ultimately resulted in the increased heterogeneity of the investigated patient population and the obtained data could only be analyzed using much more advanced, computationally demanding, and skill-intensive statistical (modeling) methods. Based on the experiences available from CT centers, if treatment protocols based on strict rules are available, they make the decision-making processes easier for both the practicing oncologists and the patients. The strict rules must also include what conditions (imaging studies, laboratory tests, etc.) the given therapy is based on. The second most influential factor affecting the treatment of patients was the various socioeconomic status of the patients. All patients receiving integrative IMT at the Dr. Kleef Medical Center had higher socioeconomic status than average, including a better financial situation and family support. Most off-label treatments offered by IOPs are expensive and not covered by public and/or private health insurance. Patients often must travel abroad and stay for a longer period to achieve optimal/effective treatment, and regular help/support from family and/or friends during those trips/treatments is essential.

It must be mentioned though, that, naturally, not everything is black and white in the case of CT either. Since the beginning of their use, it is a known fact that they have many side effects, including but not limited to alopecia, mucositis, myelosuppression, gastrointestinal side-effects such as nausea, vomiting, and diarrhea, fatigue, sterility, infertility, infusion reactions, and increased risk to infections^42^. Due to the strict regulations and guidelines patients have significantly less input and/or decision-making opportunities about their therapy, and for the same reason, the therapeutic agents the practicing oncologist can use are more limited compared to that of the IOPs.

### Limitations

Limitations of our study include the retrospective design, the high heterogeneity of patients, and the small sample sizes. The biasing effect of heterogeneity was somewhat reduced by using various robust statistical techniques, such as adjusting the baseline hazards in the survival models or performing comparisons with different matching ratios. E.g., by using these two techniques alone, we were able to significantly reduce the resulting variance that originated from the non-uniform tumor cohorts, the differences between the two main populations, and selection biases. Due to the heterogenous and low number of cases in the IMT cohort statistical significance might be biased in some comparisons, however, as previously said, the use of robust methods and consequent results from all models strengthens all tendencies that were found. It must be noted, that due to the extremely high price of nivolumab/ipilimumab, the number of patients who could afford to buy them at the IOP was even lower, which was the reason behind the small sample size of patients receiving IMT. The socioeconomic status of the two centers was significantly different, which might further affect the heterogeneity of patients. To counterbalance these effects, during the comparison of IMT and CT, propensity score matching was used to reduce confounding effects as much as possible, and baseline hazard correction was always used in all survival models when comparing the two main cohorts.

## Conclusion

Summarizing the results of the current study, a retrospective cohort analysis was performed to compare complex integrative IMT and CT with the participation of an IOP and a conventional oncology center. It was found that, except for mEHT, IMTs have mild effect on patient survival in most gastrointestinal cancer cases, compared to that of CT. However, the superiority of IMT, mainly mEHT, seemed to appear in PC. This latter observation needs further confirmation as this patient population has a poor prognosis in all aspects. To our knowledge, this is the first occasion to report a positive effect of immunomodulation in pancreatic cancer. In addition, the following general observations were made: The lack of detailed/strict protocols at the IOPs makes the analysis of scientific data significantly more challenging. The development of standardized guidelines for IOPs is strongly recommended, which should be influenced less by patient preference. Moreover, these newly developed protocols should rely on evidence-based results, imaging studies, and laboratory results that have proper control and/or normal ranges. Of course, it is not only the IOPs that need to change, but we also encourage conventional oncology centers to use off-label drugs more often if they have the opportunity, and creating a more patient-oriented environment is also extremely important. We also recommend the development of complex oncology treatment programs involving psychologists, dieticians, physiotherapists, and other specialists in addition to the oncologist, ultimately improving the quality of life of patients.

## Materials and methods

### Ethics approval

The research was approved by the Regional and Institutional Committee of Science and Research Ethics, Semmelweis University. The prospective and retrospective data collection on patients treated with concomitant mEHT was granted on February 16, 2017 (SE TUKEB 8/2017) and renewed on January 9, 2023 (SE TUKEB 8–1/2017). The retrospective data collection on colorectal cancer patients was granted on June 9, 2015 (SE TUKEB 133/2015) and on February 23, 2021 (SE TUKEB 21–14/1994). Patient consent at Semmelweis University was waived due to the retrospective, anonymized design of the study, while all patients treated at the Dr. Kleef Medical Center signed an informed consent form for future analysis of their anonymized data for research purposes. The consent patients signed also included that the Dr. Kleef Medical Center may anonymously transmit treatment data to cooperating third parties for research purposes. The research was conducted in accordance with the regulations of the WMA Declaration of Helsinki and the General Data Protection Regulation issued by the European Union.

### Patients and study design

A retrospective pilot study was conducted with the inclusion of the following three cohorts: 1.) 49 gastrointestinal tumor patients, who received IMT including nivolumab and/or ipilimumab at the Dr. Kleef Medical Center, Vienna, Austria, between 2015 and 2021. 2.) 78 pancreatic cancer (PC) patients treated at the Division of Oncology, Department of Internal Medicine and Oncology, Semmelweis University, Budapest, Hungary, between 2015 and 2019, and 3.) 835 colorectal cancer (CRC) patients, who attended the Department of Internal Medicine and Hematology, Semmelweis University, Budapest, Hungary, and at the Division of Oncology, Department of Internal Medicine and Oncology, Semmelweis University, Budapest, Hungary between 2006 and 2018. Detailed inclusion and exclusion criteria of the two non-IMT treated cohorts were further detailed in^31^ and in^43^. All patients included in the study had an Eastern Cooperative Oncology Group (ECOG) performance status score ≤ 2.

Patients of the second and third cohorts were used for propensity score-based matching. Matching was based on age, sex, staging data (only for CRC), location of the tumor, and when metastases developed (synchronous vs. metachronous). For the comparison with the 14 IMT PC patients, 14–14 PC patients treated with conventional therapeutic options with and without concomitant mEHT treatment were selected from the second cohort. 21, 42, 105, and 210 CRC patients of the third cohort were compared with the 21 IMT CRC patients after 1:1, 2:1, 5:1 and 10:1 matching, respectively. The latter was possible due to the larger sample size (*N* = 835) of the non-IMT CRC cohort. This technique was used to reduce the selection biasing effects of propensity score matching. Bias might arise after the selection of the matched pairs, as the non-observed samples might still be systematically different between the two groups. Therefore, performing the pairing with different one-to-many ratios can reduce the effect of selection bias without losing the optimal matching balance^44^. Balance statistics were determined for all matching ratios, and a slight imbalance was only observed in the case of the 1:10 pairs, where the non-IMT patients were older.

### Description of the immunomodulatory treatments

The IMT modalities were used in different combinations, and their selection was determined as described previously^20^. In brief, next-generation sequencing analysis on tumor biopsies, circulating tumor cell assays, and tumor chemosensitivity assays were used. The following therapeutic options were available:

- Low-dose ICI therapy: 0.3 mg/kg ipilimumab (anti-cytotoxic T-lymphocyte-associated protein 4) plus 0.5 mg/kg nivolumab (anti-programmed cell death protein 1) with 3 × 250 mL 2% taurolidine solution (equivalent of 15 g taurolidine). In the case of contraindication, such as ECOG > 1 or abnormal laboratory findings, ipilimumab or nivolumab monotherapy was used.
- Locoregional mEHT using either the Oncotherm EHY2000 (Oncotherm Kft., Budaörs, Hungary) or the Synchrotherm RF 600 T (Synchrotherm di Rolando Susanna, Vigevano, Italy) devices for 60 min.
- Water-filtered infrared-A hyperthermia (wIRA) using the Iratherm 1000 (Von Ardenne Institute of Applied Medical Research GmbH, Dresden, Germany) device.
- WBH uses the Heckel-HT2000 and the Heckel-HT3000 (Heckel Medizintechnik GmbH, Esslingen am Neckar, Germany) WBH devices. Based on the temperature reached during the treatment and the time spent in the device, three treatment variants can be distinguished: mild WBH (< 38.5 °C for up to 2 h), moderated WBH (38.5 °C–40.5 °C between 2 and 4 h), and long duration moderate WBH (38.5 °C–40.5 °C for up to 8 h). Furthermore, long-duration moderate WBH was performed under light sedation, and 300 mg/m^2^ cyclophosphamide infusion was also administered.
- Artificial fever therapy: using interleukin-2 (Proleukin®) with 2 × 250 mL 2% taurolidine solution (equivalent of 10 g taurolidine, to mitigate the possible cytokine storm induced by interleukin-2^45^). A total dosage of 5–14 million IU/m^2^ interleukin-2 was applied via a motor-syringe pump to reach a maximum fever temperature of 38.5 °C. Continuous body core temperature, blood pressure, heart rate and oxygen saturation monitoring was performed.
- Ozone therapy was administered using the HAB HERRMANN Hyper Medozon comfort (HERRMANN Apparatebau GmbH, Elsenfeld, Germany) device.
- Metronomic chemotherapy: a weekly dose of, e.g., 500 mg/m^2^ gemcitabine, or GemTaxol (500 mg/m^2^ gemcitabine + 60 mg/m^2^ paclitaxel). The chemotherapy agents were selected according to international guidelines.
- Other intravenous drugs:
  o High-dose vitamin C therapy: 0.5 g/kg vitamin C (capped at a total dose of 37.5 g per treatment) with 400 mg magnesium and 600 mg α-lipoic acid.
  o Curcumin: 150 mg in 500 mL 0.9% saline solution.
  o Dichloroacetate: 25 mg/kg in 500 mL 0.9% saline solution over 60 min.
  o Artesunate: 250 mg in 500 mL 0.9% saline solution over 90 min.
- Recombinant interferon-γ 1b (Imukin®): 0.5 × 10^6^ IU, administered subcutaneously.

### Details of the conventional treatments

Conventional chemo(radio)therapy (CT) was based on national and ESMO guidelines. For CRC, local radiotherapy (only for rectal cancer, if feasible and needed); a cytotoxic doublet with or without a biological agent (bevacizumab or anti-EGFR recombinant chimeric monoclonal antibody) as the first-line and second-line treatment; and irinotecan + cetuximab and regorafenib or trifluridine/tipiracil as third-line or above was administered^46–48^. For PC, radiotherapy (if feasible and needed), gemcitabine, gemcitabine + nab-paclitaxel, or the 5-fluorouracil (5-FU) + irinotecan + oxaliplatin (FOLFORINOX) regimens were used^49^.

### Clinicopathological data

Medical history data including co-morbidities and recent medications were collected. Staging of the tumors was given by histopathological examination of surgical specimens and imaging studies; the 8^th^ edition of the American Joint Committee on Cancer Staging was used^50^. Detailed location of the tumors was recorded for PC and CRC only, moreover, in CRC the sidedness of the tumor was defined as previously described^51^. Overall survival of patients was calculated from the diagnosis of the tumor until the death of the patient or until the termination of data collection (October 31, 2022). Patients alive at the time were right-censored.

### Statistical analysis

Statistical analyses were performed within the R for Windows version 4.2.2 environment (R Foundation for Statistical Computing, 2022, Vienna, Austria). Matching of the cohorts was performed via propensity score matching (R-package “Matching” version 4.10-8). Cohort comparisons were performed using Welch two sample t-tests, Fisher exact tests and Cochran-Mantel–Haenszel tests. Survival data of the cohorts were compared using “simple” and extended Cox survival models with time-dependent coefficients (R package “survival” version 3.4-0). To reduce the heterogeneity of the data, which could bias the model results, stratification was used to harmonize baseline hazards. If proportionality was violated, the survival models were extended with step functions, as described by Therneau et al.^52^. Naïve Kaplan–Meier curves were drawn with the R-package “survminer” (version 0.4.9). *P* < 0.05 was considered statistically significant. Survival, continuous, and count data were expressed as the hazard ratio (HR) with a 95% confidence interval (95% CI), the mean ± standard deviation, and the number of observations (percentage), respectively.

### Ethics declarations

The study was approved by the Regional and Institutional Committee of Science and Research Ethics, Semmelweis University (SE TUKEB 133/2015, approval date: June 9, 2015; SE TUKEB 8/2017, approval date: February 16, 2017; SE TUKEB 21–14/1994, approval date of latest modification: February 23, 2021; and SE TUKEB 8–1/2017, approval date of latest modification: January 9, 2023).

### Consent to participate

Patient data were retrieved anonymously in a retrospective manner. At the Dr. Kleef Medical Center, all patients signed informed consent to the off-label treatment they received including consent to evaluate their data retrospectively for scientific publication. At Semmelweis University, due to the retrospective design of the study, signed informed consent was waived given the anonymized, de-identified data, after the approval of the Regional and Institutional Committee of Science and Research Ethics, Semmelweis University.

### Supplementary Information

Supplementary Information.

## Data Availability

The datasets generated during and/or analyzed during the current study are available from the corresponding author on reasonable request.
