# Author Correction: Comparison of the effectiveness of integrative immunomodulatory treatments and conventional therapies on the survival of selected gastrointestinal cancer patients

**DOI:** 10.1038/s41598-024-51693-5

**Published:** 2024-01-11

**Authors:** Ralf Kleef, Magdolna Dank, Magdolna Herold, Emese Irma Agoston, Julia Lohinszky, Emoke Martinek, Zoltan Herold, Attila Marcell Szasz

**Affiliations:** 1 Dr. Kleef Medical Center, 1030 Vienna, Austria; 2 Division of Oncology, Department of Internal Medicine and Oncology, Semmelweis University, Budapest, 1082 Hungary; 3 Department of Internal Medicine and Hematology, Semmelweis University, Budapest, 1088 Hungary; 4 Department of Surgery, Transplantation and Gastroenterology, Semmelweis University, Budapest, 1082 Hungary

Correction to: *Scientific Reports* 10.1038/s41598-023-47802-5, published online 21 November 2023

The original version of this Article contained an error in Figure 3, where the ‘3 pancreatic cancer sub-cohorts’ did not display correctly.

**Figure 3:**
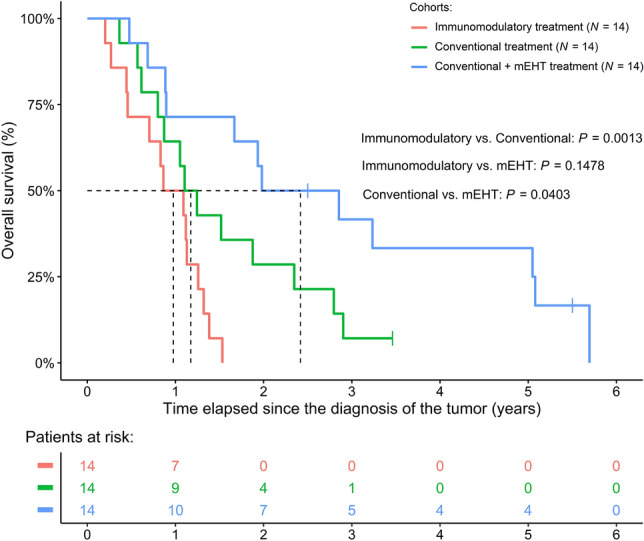
Survival difference of pancreas cancer patients between the immunomodulatory treatment and matched control cohorts with and without modulated electro-hyperthermia (mEHT) treatment. It has to be noted that naïve Kaplan–Meier curves were drawn on the figure, while the *P*-values were obtained using baseline hazard adjusted Cox regression models. Baseline hazard adjustment was performed due to the fact, that in the “Conventional” and “Conventional + mEHT” cohort the number of patients with inoperable pancreatic tumors were higher.

The original Article has been corrected.

