# Supplementary material for: Comparison of the effectiveness of integrative immunomodulatory treatments and conventional therapies on the survival of selected gastrointestinal cancer patients
Source: Sci Rep. 2023 Nov 21;13:20360. doi: 10.1038/s41598-023-47802-5 (PMC10663566; doi:10.1038/s41598-023-47802-5)
Supplement: Supplementary file 1 — Supplementary Information. [file 41598_2023_47802_MOESM1_ESM.pdf]

Comparison of the effectiveness of integrative immunomodulatory treatments and conventional therapies on the survival of selected gastrointestinal cancer patients

Ralf Kleef [†], *Magdolna Dank, Magdolna Herold, Emese Irma Agoston, Julia Lohinszky, Emoke Martinek, Zoltan Herold & Attila Marcell Szasz*

Table S1 Anamnestic data of colorectal cancer (CRC) patients receiving immunomodulating therapy (IMT), and 1:1, 2:1, 5:1 and 10:1 age, sex, side and metastasis matched CRC patients receiving conventional treatment only. Continuous and count data are presented as the mean ± standard deviation and the number of observations, respectively.

**Parameter**	**IMT (N = 21)**	**1:1 (N = 21)**	**2:1 (N = 42)**	**5:1 (N = 105)**	**10:1 (N = 210)**
Age (year)	49.00 ± 15.68	49.78 ± 12.98	51.73 ± 12.62	54.90 ± 10.85	59.48 ± 10.94
Sex (male : female)	7 : 14	8 : 13	14 : 28	46 : 59	107 : 103
TNM					
- T: 1/2/3/4/irresectable	2/2/8/7/2	2/0/11/8/0	4/2/19/14/3	5/11/53/29/7	10/26/108/45/21
- N: 0/1/2/3/irresectable	3/7/9/2	3/7/11/0	7/16/16/3	23/34/41/7	48/71/70/21
Location of the tumor					
- Cecum	2	1	4	10	19
- Ascending colon	2	2	5	12	23
- Transverse colon	1	0	1	7	11
- Descending colon	2	1	2	4	11
- Sigmoid colon	6	7	15	27	60
- Rectum	8	10	15	45	86
Sidedness					
- Left-sided	16	18	32	76	157
- Right-sided	5	3	10	29	53
Metastasis: synchronous / metachronous	11/9	11/8	21/17	58/41	127/69
Median survival since tumor diagnosis (month)	48.99	34.40	38.90	37.85	39.66
Survival rate since tumor diagnosis					
- 1-year (%)	100	100	100	100	100
- 2-year (%)	85.7	76.2	76.2	76.2	74.8
- 3-year (%)	61.5	47.6	52.4	51.4	53.3
- 4-year (%)	50.3	28.6	38.1	34.0	41.9
- 5-year (%)	22.4	19.0	24.3	29.9	33.4

TNM: Tumor – Node – Metastasis staging system.

Table S2 *p*-Values of the comparisons of the anamnestic data of colorectal cancer (CRC) patients receiving immunomodulating therapy (IMT), and 1:1, 2:1, 5:1 and 10:1 age, sex, side and metastasis matched CRC patients receiving conventional treatment only.

**Parameter**	**IMT vs 1:1**	**IMT vs 2:1**	**IMT vs 5:1**	**IMT vs 10:1**	**CMH-test**
Age (year)	0.8608 [a]	0.4929 [a]	0.1125 [a]	0.0067 [a]	–
Sex	1.0000 [b]	1.0000 [b]	0.4705 [b]	0.1693 [b]	0.1522
TNM					
- Stage T	0.4209 [b]	0.9300 [b]	0.6654 [b]	0.4894 [b]	0.4454
- Stage N	0.7518 [b]	0.9770 [b]	0.8259 [b]	0.7791 [b]	0.6200
Location of the tumor	0.9498 [b]	0.9530 [b]	0.9001 [b]	0.9556 [b]	0.7062
Sidedness	0.6965 [b]	1.0000 [b]	0.7984 [b]	0.5478 [b]	0.6448
Metastasis	1.0000 [b]	1.0000 [b]	0.9228 [b]	0.7047 [b]	0.5280
Median survival since tumor diagnosis (month)	0.4710 [c]	0.9830 [c]	0.9050 [c]	0.8980 [c]	–
Survival rate since tumor diagnosis	0.4658 [d]	0.9766 [d]	0.9107 [d]	0.9020 [d]	–

[a] Welch test; [b] Fisher’s exact test; [c] Cox regression; [d] log-rank test; CMH-test: Cochran-Mantel-Haenszel test; TNM: Tumor – Node – Metastasis staging system.

Table S3 Anamnestic data of pancreatic cancer (PC) patients receiving immunomodulating therapy (IMT), and age, sex, tumor location and metastasis matched PC patients receiving conventional treatment with or without modulated electro-hyperthermia (mEHT). Continuous and count data are presented as the mean ± standard deviation and the number of observations, respectively.

Parameter	IMT (N = 14)	Conventional (N = 14)	Conventional + mEHT (N = 14)
Age (year)	55.83 ± 9.61	58.76 ± 7.53	59.64 ± 7.99
Sex (male : female)	5 : 9	4 : 10	4 : 10
Location of the tumor			
- Head	7	8	7
- Body	4	3	3
- Tail	3	3	4
Primary tumor irresectable	7	12	12
Metastasis: synchronous / metachronous	8/5	8/4	9/2
Median survival since tumor diagnosis (month)	28.99	11.71	14.08
Survival rate since tumor diagnosis			
- 1-year (%)	71.4	50.0	64.3
- 2-year (%)	50.0	0	28.6
- 3-year (%)	41.7	0	7.1

Table S4 *p*-Values of the comparisons of the anamnestic data of pancreatic cancer (PC) patients receiving immunomodulating therapy (IMT), and age, sex, tumor location and metastasis matched PC patients receiving conventional treatment with or without modulated electro-hyperthermia (mEHT).

Parameter	IMT vs. Conventional	IMT vs. Conventional + mEHT	CMH-test
Age (year)	0.3781 [a]	0.2649 [a]	–
Sex	1.0000 [b]	1.0000 [b]	0.7774
Location of the tumor	1.0000 [b]	1.0000 [b]	0.8257
Primary tumor irresectable	0.1032 [b]	0.1032 [b]	0.0114
Metastasis	1.0000 [b]	0.3179 [b]	0.3248
Median survival since tumor diagnosis (month)	0.0004 [c]	0.0462 [b]	–
Survival rate since tumor diagnosis	0.0002 [d]	0.0546 [d]	–

[a] Welch test; [b] Fisher's exact test; [c] Cox regression; [d] log-rank test; CMH-test: Cochran-Mantel-Haenszel test.

Literature data about the potential immunomodulators

Based on *in vitro* and animal model data, artesunate, curcumin, dichloroacetate, high-dose vitamin C, interferon-γ, and interleukin-2 have known anti-tumor effects. Without going into details, they can induce apoptosis or ferroptosis, inhibit tumor cell growth, suppress angiogenesis, inflammation and oxidative stress, reverse drug resistance, downregulate immunosuppression, and induce immune cell activity against cancer cells[1-7]. Moreover, it has been reported that a high dose of vitamin C in combination with anti-PD-1 checkpoint inhibition can increase cytotoxic T cell and NK cell activity in a lymphoma mouse model[8]. However, despite the wide knowledge regarding the effects of these treatment options on cancer cells and animal models, clinical study results are extremely limited, and largely only a single agent was used rather than any combinations.

Artesunate has been investigated in colorectal cancer (CRC) only: In a small-sized randomized trial, after a two-week pre-operative treatment with daily 200 mg *per os* artesunate, decreased Ki67 activity of the tumor has been found, and less recurrence has occurred compared to that of the placebo group (1 *vs*. 6)[9]. Similarly, a single study of CRC patients is available only in the case of interferon-γ, where it was concomitantly used with fluorouracil (5-FU) and interferon-α resulting in improved response rates, but with an increased 5-FU clearance[10]. Moreover, a limited number of data is available on dichloroacetate: 1-1 cholangiocellular cancer (CCC)[11], CRC[12], and gastric cancer (GC)[13] cases with prolonged stable disease after the introduction of dichloroacetate have been reported. No clinical results on gastrointestinal tumors could be found about ozone therapy[14].

Curcumin has been tested recently as a possible candidate in preventing the development of CRC[15,16], it is safe and well-tolerated by CRC patients[17-20], and it is able to reduce the side-effects of chemo(radio)therapy and improve patient's QoL[21,22]. Overall survival of metastatic CRC patients receiving FOLFOX (folinic acid + 5-FU + oxaliplatin) plus daily 2 g oral curcumin has improved, compared to those having FOLFOX only, without any difference in the progression-free survival[20]. It has to be noted that in another study[17], no difference in tumor response could be found. Moreover, curcumin has been found to be a potential booster for more effective immunotherapy[15], and there are a few randomized trials currently running, of which the results are eagerly awaited[16,23]. In pancreatic cancer (PC), poor bioavailability, even for the high, daily 12 g oral dose, and a high rate of serious adverse events caused by curcumin could have been reported[24,25]. The introduction of nanoparticle-coupled curcumin called

Theracurmin® and other newer curcumin analogs have shown more promising results[24-26], but the biological efficacy of curcumin is still low and the available clinical data is still scarce[27]. The same applies to GC: to date, no effective form of curcumin could have been found[28], and there is no clinical data on esophageal cancer (EC)[29] and CCC.

In the early '90s – including but not limited to the studies of Akiyoshi *et al.*[30], Atzpodien *et al.*[31,32], de Braud *et al.*[33], Lygidakis *et al.*[34], and Ridolfi *et al.*[35] –, subcutaneous and intravenous interleukin-2 have been tested in combination with standard chemotherapy regimens showing little improvements compared to treatments without interleukin-2. Later, in advanced and/or metastatic CRC, phase II and III studies investigated the efficacy and safety of the GOLFIG (gemcitabine + oxaliplatin + folinic acid + 5-FU followed by subcutaneous granulocyte-macrophage colony-stimulating factor + interleukin-2)[36-38] and the GILFICet (gemcitabine + irinotecan + folinic acid + 5-FU + cetuximab followed by subcutaneous interleukin-2)[39] chemoimmunotherapy regimens, and have found improved long-term progression-free survival, overall survival[36,37] and the treatment-related immunogenicity has been associated with increased antitumor activity[38,39]. A similar observation was confirmed in PC: neo- and adjuvant chemoimmunotherapy (gemcitabine + carboplatin + mitoxantrone + interleukin-2 + interferon-γ)[40,41] in combination with resective or palliative surgery have resulted in better and long-lasting response to treatment[41] and prolonged survival of patients for both types of surgery, compared to those with surgery alone[40,41].

High-dose vitamin C can reduce the inflammation in cancer patients[42], and its effect in combination with standard chemotherapy on CRC[43-46], PC[46-49], and GC[43] have been investigated in randomized trials. In a phase I study it was found that in terminal cancer patients disease progression had occurred on average after 8 weeks when administering a daily dose of 150 to 710 mg/kg vitamin C[50]. In advanced and metastatic CRC patients, no difference in response rates, overall-, and progression-free survival could have been justified[43-45]. In contrast, patients with RAS mutation have had better survival results if treated with high-dose vitamin C + FOLFOX with or without bevacizumab[44]. In metastatic PC, in combination with gemcitabine and erlotinib, the high-dose vitamin C treatment could have reduced the size of the tumor[47], and in combination with gemcitabine 3 months and 13 – 15.1 months progression-free and overall median survival have been reported, respectively[48,49].

The United States Food and Drug Administration (FDA) approved the use of 3 mg/kg nivolumab (normal dose) plus 1 mg/kg ipilimumab (low-dose) in CRC and in renal cancer, while a reversed dosing (1 mg/kg nivolumab + 3 mg/kg ipilimumab) was allowed for melanoma and hepatocellular carcinoma[51]. However, to date, the use of low-dose nivolumab (0.5 mg/kg)

in combination with low-dose ipilimumab (0.3 mg/kg) in the routine conventional oncological practice of any cancer is uncommon[51,52]. Some data are available from a limited number of phase I and II randomized clinical trials and from observational studies[51-54]: lower doses have been reported to have less toxicity, but the efficacy of the reduced doses is largely unknown. *In vitro*, animal models, and a few clinical studies have suggested that curcumin[55], dichloroacetate[56], high dose vitamin C[8,54,57], hyperthermia[54,58], interferon-γ[59], and interleukin-2[5,54,60] can/may increase the antitumor effects of ICIs, therefore, conducting randomized trials on the subject is urgently needed.

Studies investigating metronomic chemotherapy in gastrointestinal cancers have significantly emerged in recent years[61,62]. Although the toxicity of chemotherapeutic drugs improves during metronomic chemotherapy[61,62], the results of clinical studies are controversial. The most promising results have been found in PC[61,63], while the data on GC and EC is not conclusive[61,64]. In CRC, progression-free survival usually improves but no improvement in overall survival could have been justified[61,62,65], and there is virtually no data about CCC[61]. Similar to that of low-dose ICIs, randomized trials are required to fully investigate the effectiveness of metronomic chemotherapy in these tumors.

Whole-body hyperthermia (WBH) was investigated among the earliest studies of oncologic hyperthermia[66,67]. In most early studies, the number of cases was low, results have shown that WBH is a safe and effective treatment option[67], which can also improve the quality of life of patients[67,68], but in several cases, populations with mixed tumors were analyzed[68-72]. In addition to the improved disease response[73], WBH can prolong pain control for a longer duration in CRC/rectal cancer[74]. Moreover, post-treatment carcinoembryonic antigen (CEA) can be significantly lowered one month after WBH treatment in combination with ^{131}I anti-CEA monoclonal antibody[75]. Administering WBH preoperatively can improve the postoperative reactions of the immune system[76], while no conclusive data on systemic cancer multistep therapy (WBH plus induced hyperoxemia and hyperglycemia) could have been justified[77]. Improved disease response and prolonged survival have been also confirmed in GC[78] and in PC[79]. In the GC article[78], the authors could have also described improved Karnofsky Performance Status scores, decreasing the size of hepatic metastases, and decreasing the number of abdominal lymph nodes, and reduced rate of ascites development in the treatment arm[78].

The latest advancement in oncological hyperthermia is mEHT, which was developed later, and most data are available from the last decade[66,67,80]. In the studies of Fiorentini *et al.*[81,82], mEHT-treated PC patients had almost twice as long survival compared to those receiving

chemotherapy only. This strong observation could not be verified in a previous study conducted by our team[83]. Moreover, mEHT-treated PC patients having no metastasis and/or ascites benefit more from mEHT[83], and significantly better responses to treatment have also been described previously[81,82]. In addition, our team was able to justify, that the median overall survival time significantly improves in those PC patients, who receive more mEHT treatments, compared to matched non-mEHT treated PC patients[84]. Similar results have been described in three additional studies[85-87], where no comparison to control patients has been performed, but PC patients have had a better response to treatment and longer survival than normally expected[85-87]. In rectal cancer, preoperative concomitant mEHT has been associated with T- and N-downstaging[88-91] but with controversial survival data[90,91]. To our knowledge, only a few studies/case reports are available in CCC[92], CRC[93], EC[94], and GC[95], all with favorable responses to the treatment.

References:

1 Augustin, Y., Staines, H. M. & Krishna, S. Artemisinins as a novel anti-cancer therapy: Targeting a global cancer pandemic through drug repurposing. *Pharmacol Ther* **216**, 107706, doi:10.1016/j.pharmthera.2020.107706 (2020).

2 Giordano, A. & Tommonaro, G. Curcumin and Cancer. *Nutrients* **11**, doi:10.3390/nu11102376 (2019).

3 Tataranni, T. & Piccoli, C. Dichloroacetate (DCA) and Cancer: An Overview towards Clinical Applications. *Oxid Med Cell Longev* **2019**, 8201079, doi:10.1155/2019/8201079 (2019).

4 Mussa, A. *et al.* High-Dose Vitamin C for Cancer Therapy. *Pharmaceuticals (Basel)* **15**, doi:10.3390/ph15060711 (2022).

5 Jiang, T., Zhou, C. & Ren, S. Role of IL-2 in cancer immunotherapy. *Oncoimmunology* **5**, e1163462, doi:10.1080/2162402X.2016.1163462 (2016).

6 Majidpoor, J. & Mortezaee, K. Interleukin-2 therapy of cancer-clinical perspectives. *Int Immunopharmacol* **98**, 107836, doi:10.1016/j.intimp.2021.107836 (2021).

7 Jorgovanovic, D., Song, M., Wang, L. & Zhang, Y. Roles of IFN-gamma in tumor progression and regression: a review. *Biomark Res* **8**, 49, doi:10.1186/s40364-020-00228-x (2020).

8 Luchtel, R. A. *et al.* High-dose ascorbic acid synergizes with anti-PD1 in a lymphoma mouse model. *Proc Natl Acad Sci U S A* **117**, 1666-1677, doi:10.1073/pnas.1908158117 (2020).

9 Krishna, S. *et al.* A Randomised, Double Blind, Placebo-Controlled Pilot Study of Oral Artesunate Therapy for Colorectal Cancer. *EBioMedicine* **2**, 82-90, doi:10.1016/j.ebiom.2014.11.010 (2015).

10 Grem, J. L. *et al.* A pilot study of gamma-1b-interferon in combination with fluorouracil, leucovorin, and alpha-2a-interferon. *Clin Cancer Res* **3**, 1125-1134 (1997).

11 Ishiguro, T., Ishiguro, R., Ishiguro, M. & Iwai, S. Co-treatment of dichloroacetate, omeprazole and tamoxifen exhibited synergistically antiproliferative effect on malignant tumors: in vivo experiments and a case report. *Hepatogastroenterology* **59**, 994-996, doi:10.5754/hge10507 (2012).

12 Khan, A., Marier, D., Marsden, E., Andrews, D. & Eliaz, I. A novel form of dichloroacetate therapy for patients with advanced cancer: a report of 3 cases. *Altern Ther Health Med* **20 Suppl 2**, 21-28 (2014).

13 Saito, M., Yano, K., Kamigaki, T. & Goto, S. A patient with scirrhous stomach cancer treated with combination of hyperthermotherapy and 5-aminolevulinic acid (ALA). *Anticancer Res* **33**, 2957-2963 (2013).

14 Baeza-Noci, J. & Pinto-Bonilla, R. Systemic Review: Ozone: A Potential New Chemotherapy. *Int J Mol Sci* **22**, doi:10.3390/ijms222111796 (2021).

15 Weng, W. & Goel, A. Curcumin and colorectal cancer: An update and current perspective on this natural medicine. *Semin Cancer Biol* **80**, 73-86, doi:10.1016/j.semcancer.2020.02.011 (2022).

16 Pricci, M. *et al.* Curcumin and Colorectal Cancer: From Basic to Clinical Evidences. *Int J Mol Sci* **21**, doi:10.3390/ijms21072364 (2020).

17 Greil, R. *et al.* A phase 1 dose-escalation study on the safety, tolerability and activity of liposomal curcumin (Lipocurc()) in patients with locally advanced or metastatic cancer. *Cancer Chemother Pharmacol* **82**, 695-706, doi:10.1007/s00280-018-3654-0 (2018).

18 James, M. I. *et al.* Curcumin inhibits cancer stem cell phenotypes in ex vivo models of colorectal liver metastases, and is clinically safe and tolerable in combination with FOLFOX chemotherapy. *Cancer Lett* **364**, 135-141, doi:10.1016/j.canlet.2015.05.005 (2015).

19 Sharma, R. A. *et al.* Phase I clinical trial of oral curcumin: biomarkers of systemic activity and compliance. *Clin Cancer Res* **10**, 6847-6854, doi:10.1158/1078-0432.CCR-04-0744 (2004).

20 Howells, L. M. *et al.* Curcumin Combined with FOLFOX Chemotherapy Is Safe and Tolerable in Patients with Metastatic Colorectal Cancer in a Randomized Phase IIa Trial. *J Nutr* **149**, 1133-1139, doi:10.1093/jn/nxz029 (2019).

21 Belcaro, G. *et al.* A controlled study of a lecithinized delivery system of curcumin (Meriva(R)) to alleviate the adverse effects of cancer treatment. *Phytother Res* **28**, 444-450, doi:10.1002/ptr.5014 (2014).

22 Panahi, Y. *et al.* Effects of Curcuminoids on Systemic Inflammation and Quality of Life in Patients with Colorectal Cancer Undergoing Chemotherapy: A Randomized Controlled Trial. *Adv Exp Med Biol* **1328**, 1-9, doi:10.1007/978-3-030-73234-9_1 (2021).

23 Irving, G. R. *et al.* Combining curcumin (C3-complex, Sabinsa) with standard care FOLFOX chemotherapy in patients with inoperable colorectal cancer (CUFOX): study protocol for a randomised control trial. *Trials* **16**, 110, doi:10.1186/s13063-015-0641-1 (2015).

24 Bimonte, S. *et al.* Curcumin AntiCancer Studies in Pancreatic Cancer. *Nutrients* **8**, doi:10.3390/nu8070433 (2016).

25 Kanai, M. Therapeutic applications of curcumin for patients with pancreatic cancer. *World J Gastroenterol* **20**, 9384-9391, doi:10.3748/wjg.v20.i28.9384 (2014).

26 Nagaraju, G. P., Benton, L., Bethi, S. R., Shoji, M. & El-Rayes, B. F. Curcumin analogs: Their roles in pancreatic cancer growth and metastasis. *Int J Cancer* **145**, 10-19, doi:10.1002/ijc.31867 (2019).

27 Davoodvandi, A. *et al.* Antimetastatic Effects of Curcumin in Oral and Gastrointestinal Cancers. *Front Pharmacol* **12**, 668567, doi:10.3389/fphar.2021.668567 (2021).

28 Jakubek, M. *et al.* Strategy for improved therapeutic efficiency of curcumin in the treatment of gastric cancer. *Biomed Pharmacother* **118**, 109278, doi:10.1016/j.biopha.2019.109278 (2019).

29 Wang, S. *et al.* The anticancer effects of curcumin and clinical research progress on its effects on esophageal cancer. *Front Pharmacol* **13**, 1058070, doi:10.3389/fphar.2022.1058070 (2022).

30 Akiyoshi, T. *et al.* The effect of recombinant interleukin 2 in combination with mitomycin C on advanced cancer. *Jpn J Surg* **20**, 365-368, doi:10.1007/BF02470676 (1990).

31 Atzpodien, J. *et al.* Treatment of metastatic colorectal cancer patients with 5-fluorouracil in combination with recombinant subcutaneous human interleukin-2 and alpha-interferon. *Oncology* **51**, 273-275, doi:10.1159/000227348 (1994).

32 Atzpodien, J. *et al.* Low-dose subcutaneous recombinant interleukin-2 in advanced human malignancy: a phase II outpatient study. *Mol Biother* **2**, 18-26 (1990).

33 de Braud, F., Colleoni, M., Noli, F. & Bajetta, E. Home treatment with interleukin 2 and beta-interferon in patients with colorectal cancer and hepatocellular carcinoma. *Oncology* **51**, 472-476, doi:10.1159/000227386 (1994).

34 Lygidakis, N. J. *et al.* Combined immunopharmaceutical therapy of patients with unresectable pancreatic carcinoma. *Hepatogastroenterology* **42**, 1039-1052 (1995).

35 Ridolfi, R. *et al.* A phase II study of advanced colorectal cancer patients treated with combination 5-fluorouracil plus leucovorin and subcutaneous interleukin-2 plus alpha interferon. *J Chemother* **6**, 265-271, doi:10.1080/1120009x.1994.11741163 (1994).

36 Correale, P. *et al.* Immunity feedback and clinical outcome in colon cancer patients undergoing chemoimmunotherapy with gemcitabine + FOLFOX followed by subcutaneous granulocyte macrophage colony-stimulating factor and aldesleukin (GOLFIG-1 Trial). *Clin Cancer Res* **14**, 4192-4199, doi:10.1158/1078-0432.CCR-07-5278 (2008).

37 Correale, P. *et al.* Gemcitabine, oxaliplatin, levofolinate, 5-fluorouracil, granulocyte-macrophage colony-stimulating factor, and interleukin-2 (GOLFIG) versus FOLFOX chemotherapy in metastatic colorectal cancer patients: the GOLFIG-2 multicentric open-label randomized phase III trial. *J Immunother* **37**, 26-35, doi:10.1097/CJI.0000000000000004 (2014).

38 Caraglia, M. *et al.* GOLFIG Chemo-Immunotherapy in Metastatic Colorectal Cancer Patients. A Critical Review on a Long-Lasting Follow-Up. *Front Oncol* **9**, 1102, doi:10.3389/fonc.2019.01102 (2019).

39 Botta, C. *et al.* Immune-modulating effects of the newest cetuximab-based chemoimmunotherapy regimen in advanced colorectal cancer patients. *J Immunother* **35**, 440-447, doi:10.1097/CJI.0b013e31825943aa (2012).

40 Lygidakis, N. J. *et al.* A prospective randomized study using adjuvant locoregional chemoimmunotherapy in combination with surgery for pancreatic carcinoma. *Hepatogastroenterology* **45**, 2376-2381 (1998).

41 Lygidakis, N. J. *et al.* Pancreatic resection for pancreatic carcinoma combined with neo- and adjuvant locoregional targeting immuno-chemotherapy--a prospective randomized study. *Hepatogastroenterology* **45**, 396-403 (1998).

42 Mikirova, N., Casciari, J., Rogers, A. & Taylor, P. Effect of high-dose intravenous vitamin C on inflammation in cancer patients. *J Transl Med* **10**, 189, doi:10.1186/1479-5876-10-189 (2012).

43 Wang, F. *et al.* Phase I study of high-dose ascorbic acid with mFOLFOX6 or FOLFIRI in patients with metastatic colorectal cancer or gastric cancer. *BMC Cancer* **19**, 460, doi:10.1186/s12885-019-5696-z (2019).

44 Wang, F. *et al.* A Randomized, Open-Label, Multicenter, Phase 3 Study of High-Dose Vitamin C Plus FOLFOX +/- Bevacizumab versus FOLFOX +/- Bevacizumab in Unresectable Untreated Metastatic Colorectal Cancer (VITALITY Study). *Clin Cancer Res* **28**, 4232-4239, doi:10.1158/1078-0432.CCR-22-0655 (2022).

45 Moertel, C. G. *et al.* High-dose vitamin C versus placebo in the treatment of patients with advanced cancer who have had no prior chemotherapy. A randomized double-blind comparison. *N Engl J Med* **312**, 137-141, doi:10.1056/NEJM198501173120301 (1985).

46 Stephenson, C. M., Levin, R. D., Spector, T. & Lis, C. G. Phase I clinical trial to evaluate the safety, tolerability, and pharmacokinetics of high-dose intravenous ascorbic acid in patients with advanced cancer. *Cancer Chemother Pharmacol* **72**, 139-146, doi:10.1007/s00280-013-2179-9 (2013).

47 Monti, D. A. *et al.* Phase I evaluation of intravenous ascorbic acid in combination with gemcitabine and erlotinib in patients with metastatic pancreatic cancer. *PLoS One* **7**, e29794, doi:10.1371/journal.pone.0029794 (2012).

48 Polireddy, K. *et al.* High Dose Parenteral Ascorbate Inhibited Pancreatic Cancer Growth and Metastasis: Mechanisms and a Phase I/IIa study. *Sci Rep* **7**, 17188, doi:10.1038/s41598-017-17568-8 (2017).

49 Welsh, J. L. *et al.* Pharmacological ascorbate with gemcitabine for the control of metastatic and node-positive pancreatic cancer (PACMAN): results from a phase I clinical trial. *Cancer Chemother Pharmacol* **71**, 765-775, doi:10.1007/s00280-013-2070-8 (2013).

50 Riordan, H. D. *et al.* A pilot clinical study of continuous intravenous ascorbate in terminal cancer patients. *P R Health Sci J* **24**, 269-276 (2005).

51 Jiang, M., Hu, Y., Lin, G. & Chen, C. Dosing Regimens of Immune Checkpoint Inhibitors: Attempts at Lower Dose, Less Frequency, Shorter Course. *Front Oncol* **12**, 906251, doi:10.3389/fonc.2022.906251 (2022).

52 Renner, A., Burotto, M. & Rojas, C. Immune Checkpoint Inhibitor Dosing: Can We Go Lower Without Compromising Clinical Efficacy? *J Glob Oncol* **5**, 1-5, doi:10.1200/JGO.19.00142 (2019).

53 Barbee, M. S., Ogunniyi, A., Horvat, T. Z. & Dang, T. O. Current status and future directions of the immune checkpoint inhibitors ipilimumab, pembrolizumab, and nivolumab in oncology. *Ann Pharmacother* **49**, 907-937, doi:10.1177/1060028015586218 (2015).

54 Kleef, R. *et al.* Low-dose ipilimumab plus nivolumab combined with IL-2 and hyperthermia in cancer patients with advanced disease: exploratory findings of a case series of 131 stage IV cancers - a retrospective study of a single institution. *Cancer Immunol Immunother* **70**, 1393-1403, doi:10.1007/s00262-020-02751-0 (2021).

55 Paul, S. & Sa, G. Curcumin as an Adjuvant to Cancer Immunotherapy. *Front Oncol* **11**, 675923, doi:10.3389/fonc.2021.675923 (2021).

56 Rostamian, H. *et al.* Restricting tumor lactic acid metabolism using dichloroacetate improves T cell functions. *BMC Cancer* **22**, 39, doi:10.1186/s12885-021-09151-2 (2022).

57 Bedhiafi, T. *et al.* The potential role of vitamin C in empowering cancer immunotherapy. *Biomed Pharmacother* **146**, 112553, doi:10.1016/j.biopha.2021.112553 (2022).

58 Li, Z., Deng, J., Sun, J. & Ma, Y. Hyperthermia Targeting the Tumor Microenvironment Facilitates Immune Checkpoint Inhibitors. *Front Immunol* **11**, 595207, doi:10.3389/fimmu.2020.595207 (2020).

59 Ding, G. *et al.* IFN-gamma down-regulates the PD-1 expression and assist nivolumab in PD-1-blockade effect on CD8+ T-lymphocytes in pancreatic cancer. *BMC Cancer* **19**, 1053, doi:10.1186/s12885-019-6145-8 (2019).

60 Mortezaee, K. & Majidpoor, J. Checkpoint inhibitor/interleukin-based combination therapy of cancer. *Cancer Med* **11**, 2934-2943, doi:10.1002/cam4.4659 (2022).

61 Filippi, R. *et al.* Rationale for the use of metronomic chemotherapy in gastrointestinal cancer. *Expert Opin Pharmacother* **19**, 1451-1463, doi:10.1080/14656566.2018.1512585 (2018).

62 Cazzaniga, M. E. *et al.* Metronomic Chemotherapy. *Cancers (Basel)* **13**, doi:10.3390/cancers13092236 (2021).

63 Isacoff, W. H., Cooper, B., Bartlett, A., McCarthy, B. & Yu, K. H. ChemoSensitivity Assay Guided Metronomic Chemotherapy Is Safe and Effective for Treating Advanced Pancreatic Cancer. *Cancers (Basel)* **14**, doi:10.3390/cancers14122906 (2022).

64 Noronha, V. *et al.* Oral metronomic chemotherapy after definitive chemoradiation in esophageal squamous cell carcinoma: a randomized clinical trial. *Esophagus* **19**, 670-682, doi:10.1007/s10388-022-00923-8 (2022).

65 Chen, L., Cao, X., Li, J., Liu, C. & Jiang, T. Efficacy and safety of metronomic chemotherapy in maintenance therapy for metastatic colorectal cancer: A systematic review of randomized controlled trials. *Medicine (Baltimore)* **101**, e31659, doi:10.1097/MD.0000000000031659 (2022).

66 Jha, S., Sharma, P. K. & Malviya, R. Hyperthermia: Role and Risk Factor for Cancer Treatment. *Achievements in the Life Sciences* **10**, 161-167, doi:10.1016/j.als.2016.11.004 (2016).

67 Herold, Z., Szasz, A. M. & Dank, M. Evidence based tools to improve efficiency of currently administered oncotherapies for tumors of the hepatopancreatobiliary system. *World J Gastrointest Oncol* **13**, 1109-1120, doi:10.4251/wjgo.v13.i9.1109 (2021).

68 Bull, J. M. *et al.* Fever-range whole-body thermal therapy combined with cisplatin, gemcitabine, and daily interferon-alpha: a description of a phase I-II protocol. *Int J Hyperthermia* **24**, 649-662, doi:10.1080/02656730802104740 (2008).

69 Robins, H. I. *et al.* Rise in plasma beta-endorphin, ACTH, and cortisol in cancer patients undergoing whole body hyperthermia. *Horm Metab Res* **19**, 441-443, doi:10.1055/s-2007-1011847 (1987).

70 Robins, H. I. *et al.* Phase I clinical trial of carboplatin and 41.8 degrees C whole-body hyperthermia in cancer patients. *J Clin Oncol* **11**, 1787-1794, doi:10.1200/JCO.1993.11.9.1787 (1993).

71 Robins, H. I. *et al.* Phase I clinical trial of melphalan and 41.8 degrees C whole-body hyperthermia in cancer patients. *J Clin Oncol* **15**, 158-164, doi:10.1200/JCO.1997.15.1.158 (1997).

72 Kerner, T. *et al.* Whole body hyperthermia: a secure procedure for patients with various malignancies? *Intensive Care Med* **25**, 959-965, doi:10.1007/s001340050989 (1999).

73 Hegewisch-Becker, S. *et al.* Whole-body hyperthermia (41.8 degrees C) combined with bimonthly oxaliplatin, high-dose leucovorin and 5-fluorouracil 48-hour continuous infusion in pretreated metastatic colorectal cancer: a phase II study. *Ann Oncol* **13**, 1197-1204, doi:10.1093/annonc/mdf216 (2002).

74 Estes, N. C., Morphis, J. G., Hornback, N. B. & Jewell, W. R. Intraarterial chemotherapy and hyperthermia for pain control in patients with recurrent rectal cancer. *Am J Surg* **152**, 597-601, doi:10.1016/0002-9610(86)90433-2 (1986).

75 Mittal, B. B. *et al.* Phase I/II trial of combined 131I anti-CEA monoclonal antibody and hyperthermia in patients with advanced colorectal adenocarcinoma. *Cancer* **78**, 1861-1870 (1996).

76 Sulyok, I. *et al.* Effect of preoperative fever-range whole-body hyperthermia on immunological markers in patients undergoing colorectal cancer surgery. *Br J Anaesth* **109**, 754-761, doi:10.1093/bja/aes248 (2012).
77 Hildebrandt, B. *et al.* Whole-body hyperthermia in the scope of von Ardenne's systemic cancer multistep therapy (sCMT) combined with chemotherapy in patients with metastatic colorectal cancer: a phase I/II study. *Int J Hyperthermia* **20**, 317-333, doi:10.1080/02656730310001637316 (2004).
78 Zhao, C., Dai, C. & Chen, X. Whole-body hyperthermia combined with hyperthermic intraperitoneal chemotherapy for the treatment of stage IV advanced gastric cancer. *Int J Hyperthermia* **28**, 735-741, doi:10.3109/02656736.2012.734894 (2012).
79 Bakshandeh-Bath, A. *et al.* Preclinical and clinical aspects of carboplatin and gemcitabine combined with whole-body hyperthermia for pancreatic adenocarcinoma. *Anticancer Res* **29**, 3069-3077 (2009).
80 Alshaibi, H. F., Al-Shehri, B., Hassan, B., Al-Zahrani, R. & Assiss, T. Modulated Electrohyperthermia: A New Hope for Cancer Patients. *Biomed Res Int* **2020**, 8814878, doi:10.1155/2020/8814878 (2020).
81 Fiorentini, G. *et al.* Modulated Electro-Hyperthermia as Palliative Treatment for Pancreatic Cancer: A Retrospective Observational Study on 106 Patients. *Integr Cancer Ther* **18**, 1534735419878505, doi:10.1177/1534735419878505 (2019).
82 Fiorentini, G. *et al.* Modulated electro-hyperthermia in stage III and IV pancreatic cancer: Results of an observational study on 158 patients. *World J Clin Oncol* **12**, 1064-1071, doi:10.5306/wjco.v12.i11.1064 (2021).
83 Petenyi, F. G. *et al.* Modulated Electro-Hyperthermic (mEHT) Treatment in the Therapy of Inoperable Pancreatic Cancer Patients-A Single-Center Case-Control Study. *Diseases* **9**, doi:10.3390/diseases9040081 (2021).
84 Dank, M. *et al.* Electromagnetic procedures in the treatment of pancreatic cancer: eminent or resentful? *Magyar Onk* **67**, 194-201 (2023).
85 Dani, A., Varkonyi, A., Magyar, T., Kalden, M. & Szasz, A. Clinical study for advanced pancreas cancer treated by oncothermia. *Oncothermia Journal* **6**, 11-25 (2012).
86 Volovat, C., Volovat, S., Scripcaru, V. & Miron, L. Second-line chemotherapy with gemcitabine and oxaliplatin in combination with loco-regional hyperthermia (EHY-2000) in patients with refractory metastatic pancreatic cancer - preliminary results of a prospective trial. *Romanian Reports in Physics* **66**, 166-174 (2014).

87 Iyikesici, M. S. Long-Term Survival Outcomes of Metabolically Supported Chemotherapy with Gemcitabine-Based or FOLFIRINOX Regimen Combined with Ketogenic Diet, Hyperthermia, and Hyperbaric Oxygen Therapy in Metastatic Pancreatic Cancer. *Complement Med Res* **27**, 31-39, doi:10.1159/000502135 (2020).

88 You, S. H. & Kim, S. Feasibility of modulated electro-hyperthermia in preoperative treatment for locally advanced rectal cancer: Early phase 2 clinical results. *Neoplasma* **67**, 677-683, doi:10.4149/neo_2020_190623N538 (2020).

89 Kim, S., Lee, J. H., Cha, J. & You, S. H. Beneficial effects of modulated electro-hyperthermia during neoadjuvant treatment for locally advanced rectal cancer. *Int J Hyperthermia* **38**, 144-151, doi:10.1080/02656736.2021.1877837 (2021).

90 Lee, Y. *et al.* Long-Term Feasibility of 13.56 MHz Modulated Electro-Hyperthermia-Based Preoperative Thermoradiochemotherapy in Locally Advanced Rectal Cancer. *Cancers (Basel)* **14**, doi:10.3390/cancers14051271 (2022).

91 Wang, Y. *et al.* Deep regional hyperthermia combined with modern concurrent chemoradiotherapy increases T-downstaging rate in locally advanced rectal cancer. *Int J Hyperthermia* **39**, 431-436, doi:10.1080/02656736.2022.2044077 (2022).

92 Ryu, J. *et al.* Patient With Unresectable Cholangiocarcinoma Treated With Radiofrequency Hyperthermia in Combination With Chemotherapy: A Case Report. *Integr Cancer Ther* **17**, 558-561, doi:10.1177/1534735417722225 (2018).

93 Ranieri, G. *et al.* Bevacizumab-Based Chemotherapy Combined with Regional Deep Capacitive Hyperthermia in Metastatic Cancer Patients: A Pilot Study. *Int J Mol Sci* **18**, doi:10.3390/ijms18071458 (2017).

94 Sheng, L., Ji, Y., Wu, Q. & Du, X. Regional hyperthermia combined with radiotherapy for esophageal squamous cell carcinoma with supraclavicular lymph node metastasis. *Oncotarget* **8**, 5339-5348, doi:10.18632/oncotarget.14148 (2017).

95 Iyikesici, M. S. Survival outcomes of metabolically supported chemotherapy combined with ketogenic diet, hyperthermia, and hyperbaric oxygen therapy in advanced gastric cancer. *Niger J Clin Pract* **23**, 734-740, doi:10.4103/njcp.njcp_509_18 (2020).
